# Induction of Apoptosis and Cytotoxicity by Raphasatin in Human Breast Adenocarcinoma MCF-7 Cells

**DOI:** 10.3390/molecules23123092

**Published:** 2018-11-27

**Authors:** Muhammad Din Ibrahim, Saie Brindha Kntayya, Nooraini Mohd Ain, Renato Iori, Costas Ioannides, Ahmad Faizal Abdull Razis

**Affiliations:** 1 UPM-MAKNA Cancer Research Laboratory, Institute of Bioscience, Universiti Putra Malaysia, 43400 UPM Serdang, Selangor, Malaysia; marc_dean89@yahoo.com (M.D.I.); saiebrindhak@yahoo.co.uk (S.B.K.); noorainim@upm.edu.my (N.M.A.); 2 CREA Consiglio per la ricerca in agricoltura e l’analisi dell’economia agrarian, Centro di ricerca Agricoltura e Ambiente (CREA-AA), Via di Corticella 133, 40128 Bologna, Italy; renato.iori48@gmail.com; 3 Faculty of Health and Medical Sciences, University of Surrey, Guildford, Surrey GU2 7XH, UK; c.ioannides@surrey.ac.uk; 4 Laboratory of Molecular Biomedicine, Institute of Bioscience, Universiti Putra Malaysia, 43400 UPM Serdang, Selangor, Malaysia; 5 Laboratory of Food Safety and Food Integrity, Institute of Tropical Agriculture and Food Security, Universiti Putra Malaysia, 43400 UPM Serdang, Selangor, Malaysia; 6 Department of Food Science, Faculty of Food Science and Technology, Universiti Putra Malaysia, 43400 UPM Serdang, Selangor, Malaysia

**Keywords:** glucoraphasatin, raphasatin, glucosinolates, isothiocyanates, human breast adenocarcinoma cell line, cytotoxicity, apoptosis, cell cycle

## Abstract

Glucoraphasatin (GRH), a glucosinolate present abundantly in the plants of the *Brassicaceae* family, is hydrolyzed by myrosinase to raphasatin, which is considered responsible for its cancer chemopreventive activity; however, the underlying mechanisms of action have not been investigated, particularly in human cell lines. The aims of this study are to determine the cytotoxicity of raphasatin, and to evaluate its potential to cause apoptosis and modulate cell cycle arrest in human breast adenocarcinoma MCF-7 cells. The cytotoxicity was determined following incubation of the cells with glucoraphasatin or raphasatin (0–100 µM), for 24, 48, and 72 h. GRH displayed no cytotoxicity as exemplified by the 3-(4,5-dimethylthiazol-2-yl)-2,5-diphenyltetrazolium bromide (MTT) assay. When myrosinase was added to the incubation system to convert GRH to raphasatin, cytotoxicity was evident. Exposure of the cells to raphasatin stimulated apoptosis, as was exemplified by cell shrinkage, membrane blebbing, chromatin condensation, and nuclear fragmentation. Moreover, using Annexin V-FITC assay, raphasatin induced apoptosis, as witnessed by changes in cellular distribution of cells, at different stages of apoptosis; in addition, raphasatin caused the arrest of the MCF-7 cells at the G_2_ + M phase. In conclusion, raphasatin demonstrated cancer chemopreventive potential against human breast adenocarcinoma (MCF-7) cells, through induction of apoptosis and cell cycle arrest.

## 1. Introduction

Cancer is a failure of the mechanisms that regulate cell growth and division, and remains a leading cause of death worldwide. Although chemo- and radio-therapies are currently used routinely in cancer therapy [1], these are accompanied by serious side-effects, as a result of damage to normal tissues surrounding the tumors [2]. Moreover, tumors may demonstrate resistance to these treatments [3]. Breast cancer is a very common disease among Malaysian women and worldwide, with about 150–200 new cases being diagnosed, annually, in the Breast Clinic in Kuala Lumpur Hospital, alone. It is estimated that 1 in 20 women will be diagnosed with breast cancer [4,5]. Breast cancer accounted for 16.5% of all cancer cases registered with the Malaysian National Cancer Registry in 2006, and 34.4% of the registered breast cancer cases were in the working age of 15–49 years [6].

During the last few decades the importance of diet in the etiology of human cancer has been well-documented [7]. Many phytochemicals, including ascorbic acid and phenolics, such as anthocyanins and flavonoids, possess antioxidant activity and are considered important contributors to the beneficial effects linked to the consumption of vegetable-rich diets. Broccoli (*Brassica oleracea*), radish (*Raphanus sativus* L.), kale, and cabbage (*B. oleracea* L.) are examples of Brassica vegetables that are rich in these antioxidants, as well as glucosinolates, which have attracted much attention, in recent years, due to the remarkable anti-carcinogenic activity of isothiocyanates, their major hydrolysis products [8,9].

In a number of epidemiological studies, the consumption of cruciferous vegetables is associated with lower cancer incidence, at a number of sites [10,11]. The chemopreventive property of these vegetables is recognized to be due to glucosinolates, a class of sulfur-containing glycosides that are present in significant amounts [10]. Glucosinolates (GLs) are secondary metabolites of Brassica vegetables that are related to their health-promoting benefits, as their hydrolysis products, isothiocyanates, possess chemopreventive and antioxidant properties [12].

**Figure 1:**
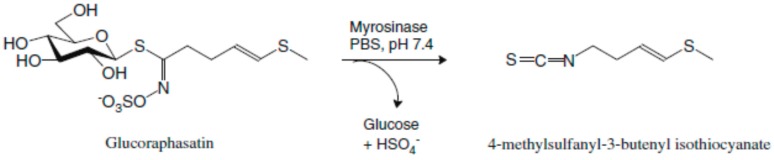
The hydrolysis process of glucoraphasatin by myrosinase produces 4-methylsulfanyl-3-butenyl isothiocyanate, raphasatin. Adapted from Razis et al. [20].

4-methylsulfanyl-3-butenyl glucosinolate, also referred to as GRH, is a glucosinolate, the major source of which is *Raphanus sativus* (Kaiware Daikon), a white radish that is extensively consumed in Japan and increasingly in Europe and North America [13,14]. *Raphanus sativus* L. radishes have lately attracted interest in the scientific community because of their health-promoting phytochemical content. The interest focused, particularly, on the isothiocyanate raphasatin (Figure 1), which is released by myrosinase hydrolysis, upon chewing, cutting, or other mechanical disruption of the fresh (uncooked) vegetable tissues [15]. Along with contributing to the distinctive pungent taste of radish, isothiocyanates are powerful indirect antioxidants possessing chemopreventive activity and potentially therapeutic activity [16].

The ability to induce apoptosis is an important property of anti-cancer candidate drugs. Many methods have been developed to evaluate the potential of chemical agents to stimulate apoptosis, including morphological studies, biochemical assays, ELISA, flow cytometry analysis and others, of which morphological studies appear to be the most reliable [17]. As previous studies have demonstrated that isothiocyanates manifest their chemopreventive activity, at least partly, by modulating favorably the processes of apoptosis and cell proliferation [18,19,20,21], the present study was undertaken to establish the potential of GRH and its degradation product raphasatin, to initiate apoptosis and cell cycle arrest in human breast adenocarcinoma (MCF-7) cells.

## 2. Results

### 2.1. Effects of GRH and Raphasatin on Cell Viability of MCF-7 Cells

In this study, the viability of MCF-7 cells was investigated, following exposure to GRH and raphasatin. At the concentrations studied (1–100 µM), GRH had no effect on the MCF-7 cells (Table 1). Conversely, its degradation product, the isothiocyanate raphasatin was cytotoxic, following incubation for 24, 48, and 72 h. Paclitaxel (0–25 nM) served as the positive control and was highly cytotoxic to the cells. 

### 2.2. Morphological Analyses of Apoptosis Using the Terminal Deoxynucleotidyl Transferase dUTP Nick End Labelling (TUNEL) Assay, Acridine Orange/Propidium Iodide (AO/PI) Staining, and 4′,6-Diamidino-2-phenylindole (DAPI) Staining

**Figure 2:**
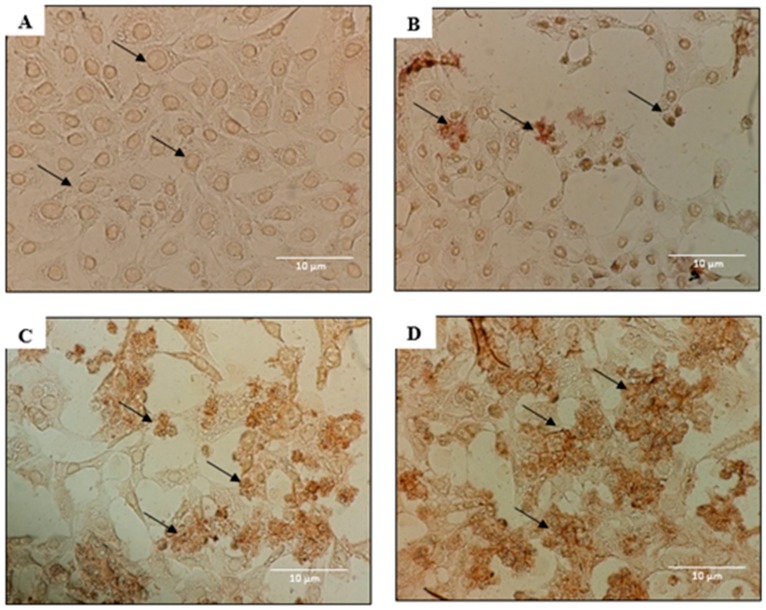
Raphasatin-mediated apoptosis in MCF-7 cells, as determined using the DeadEnd Colorimetric terminal deoxynucleotidyl transferase dUTP nick end labelling (TUNEL) assay. MCF-7 cells were treated with 9.84 µM (IC_50_) concentration of raphasatin for various periods of time. Images represent one of three independent experiments. Morphological changes after the treatment for 24 h (**B**), 48 h (**C**), and 72 h (**D**) were compared with the untreated cells (**A**), which acted as control. The arrows in **A** denote the viable unaffected cells, and in **B**, **C**, and **D** are the typical apoptotic cells (dark brown color stained in **B**, **C**, and **D**) resulting from nuclear DNA fragmentation.

In morphological studies, the TUNEL assay, AO/PI staining and DAPI staining assays were employed to assess the morphological changes in MCF-7 cells, following exposure to raphasatin, for different periods of time, namely 24, 48, and 72 h. In the TUNEL assay the untreated cells (Figure 2A) were healthy, characterized by a round-shape, and unstained by TUNEL. After treatment for 24 h (Figure 2B), the cells began to be stained with dark brown color indicating that some cells underwent apoptosis at an early stage. After a 48 h treatment (Figure 2C), the number of dark brown-stained cells increased; about 50% of the cell population had undergone apoptosis, which caused nuclear DNA fragmentation. Finally, the 72 h-treated cells exhibited the highest number of apoptotic cells, covering almost 70% of the cell population (Figure 2D).

**Figure 3:**
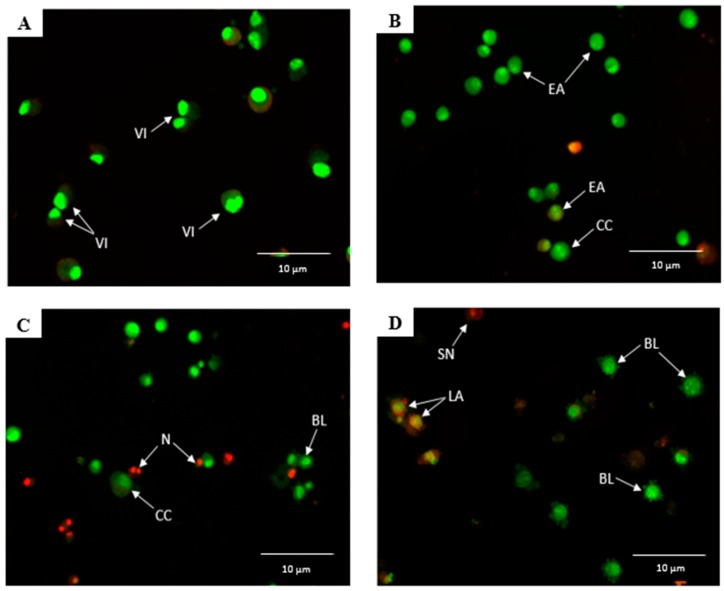
Raphasatin-induced apoptosis in the MCF-7 cells assessed by acridine orange/propidium iodide (AO/PI) staining. MCF-7 cells were treated with 9.84 µM (IC_50_) concentration of raphasatin, for 24 h (**B**), 48 h (**C**), and 72 h (**D**), and were compared with the untreated cells (**A**). Images represent one of the three independent experiments. Viable cells (VI), early apoptosis (EA), chromatin condensation (CC), blebbing (BL), late apoptosis (LA), necrosis (N), and secondary necrosis (SN).

In AO/PI staining, the untreated cells (Figure 3A) did not show any morphological changes, and only bright intact green-colored, round-shaped, viable cells were observed, indicating no signs of apoptosis. However, apoptosis was clearly evident upon 24 h (Figure 3B) treatment with raphasatin, where early apoptosis with fragmented nucleus and chromatin condensation features were evident, and the round-shaped cells also began to shrink. After the 48 h treatment (Figure 3C), apoptosis was clearly present, with membrane blebbing and chromatin condensation being visible. The blebs surrounding the single cell signified that apoptosis had developed. In addition, some necrotic cells were clearly seen, with the red-stained color cells. Cells exposed to raphasatin for 72 h (Figure 3D) exhibited more severe apoptosis, including membrane blebbing, late apoptosis, secondary necrosis, and formation of apoptotic bodies. Furthermore, in the late stages of apoptosis, changes such as the presence of reddish-orange color, due to the binding of AO to the denatured DNA, were observed after the 72 h treatment of the cells. 

**Figure 4:**
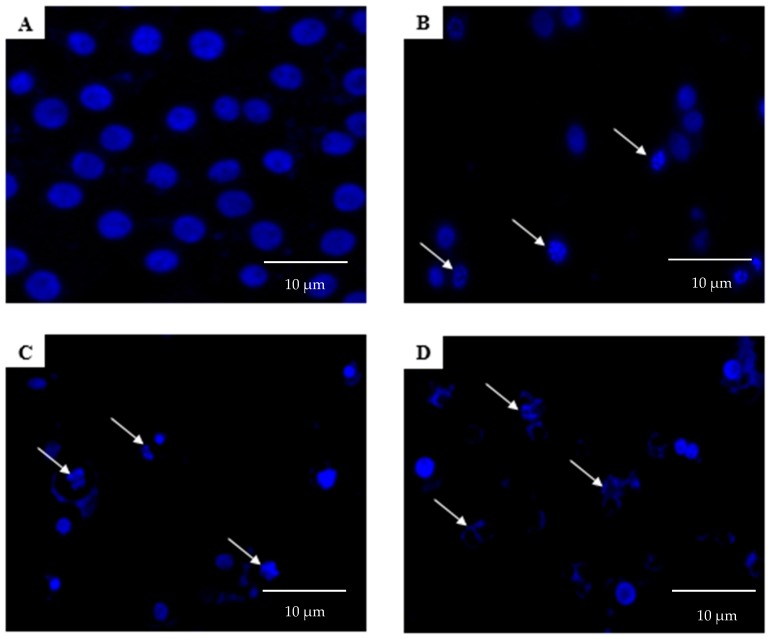
The fluorescent micrographs showing the effect of raphasatin on apoptosis, assessed by DAPI in MCF-7 cells. MCF-7 cells were treated with 9.84 µM (IC_50_) concentration of raphasatin for 24 h (**B**), 48 h (**C**), and 72 h (**D**), and compared with the untreated cells (**A**). Images represent one of the three independent experiments. The arrows in B, C, and D show the nuclear fragmentation and chromatin condensation commensurate, with apoptosis.

**Figure 5:**
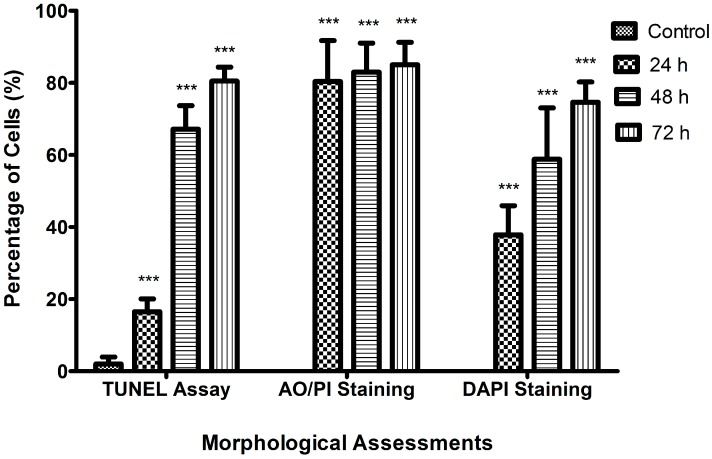
Bar graph showing the effect of raphasatin on apoptosis assessed using TUNEL, AO/PI, and DAPI in the MCF-7 cells. The cells were treated with 9.84 µM (IC_50_) concentration of raphasatin for 24 h, 48 h, and 72 h, and compared with the untreated cells. Data are expressed in mean ± standard deviation (SD). *** *p* < 0.001.

As shown in Figure 4, DAPI staining indicated that the treatment of MCF-7 cells with raphasatin, for the different periods of time (24, 48, and 72 h), induced morphological features typical of apoptotic cells, including nuclear fragmentation, micronuclei formation, and chromatin condensation, as compared with the control cells that maintained the round shape. In contrast, the treated cells exhibited the morphological characteristics of early apoptosis. Apoptotic cells and pyknotic nuclei with bright blue color intensity were noted. After the 24 h treatment with raphasatin (Figure 4B), the cells’ morphology began to alter. Some of the cells had undergone nuclear fragmentation, an indicator of apoptosis. Some viable cells were still visible as the apoptosis was at an early stage. After treatment for 48 h (Figure 4C), more fragmented and apoptotic cells were visible. These features appeared more frequently, as the duration of incubation increased to 72 h (Figure 4D), when the treated cells showed chromatin condensation indicative of late apoptosis. The normal round-shaped cells had condensed and the majority of the cells had completely undergone apoptosis. Figure 5 shows the significant differences between the control (untreated) with the treated cells at 24, 48, and 72 h of incubation for all the morphological analyses.

### 2.3. Raphasatin-Induced Apoptosis in the MCF-7 Cells Determined by Annexin V-FITC/PI Double Staining and Using Flow Cytometry

**Figure 6:**
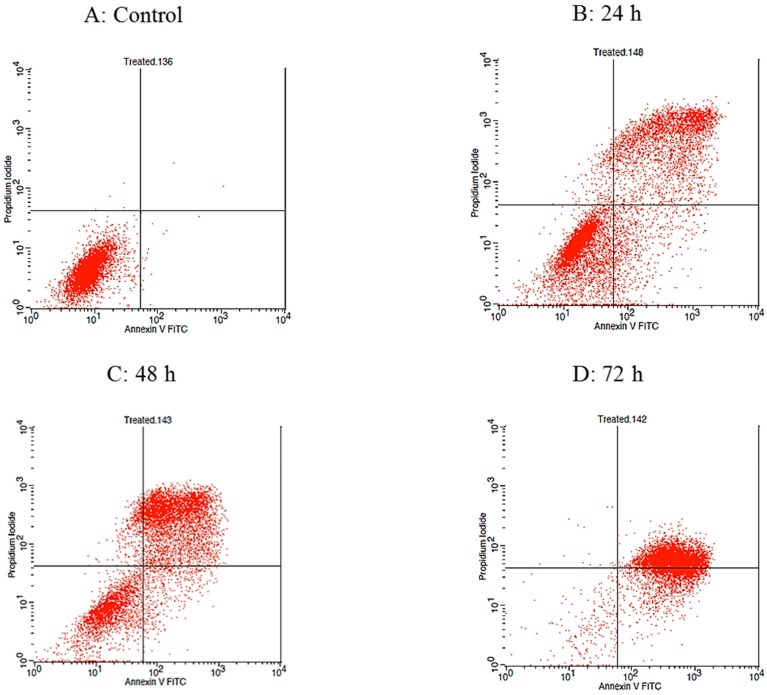
Annexin V-FITC/PI staining to evaluate apoptosis. The MCF-7 cells were treated with IC_50_ concentration of raphasatin (9.84 µM), incubated for 24 (B), 48 (C), and 72 h (D) and compared with untreated cells (**A**). The rate of apoptosis was determined by flow cytometry, using the Annexin V-FITC/PI staining assay. The data represent one of three independent experiments. (i) Lower left quadrant represents the viable cells, (ii) lower right quadrant shows the early apoptosis cells, (iii) upper right quadrant signifies the late apoptosis cells, and (iv) the upper left quadrant indicates dead cells.

The qualitative assessment of apoptosis was carried out utilizing the Annexin V-FITC/PI staining assay and using flow cytometry in order to determine the cell distribution at four different quadrants, namely viable cells, early apoptosis, late apoptosis, and necrotic cells. The histogram illustrates the time course of Annexin V-FITC/PI binding to the phosphatidylyserine (PS), in the apoptotic cells (Figure 6). 

**Figure 7:**
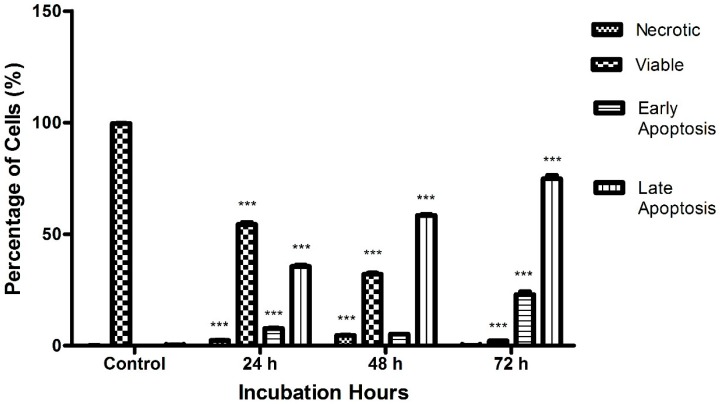
The effect of exposure time on the raphasatin-induced rate of apoptosis in the MCF-7 cells. The cells were treated with 9.84 µM (IC_50_) concentration of raphasatin for 24–72 h. The data comparing the percentage of cells between the untreated with treated, in terms of viable, early, and late apoptosis and necrotic for 24, 48, and 72 h of incubation. The data expressed as mean ± SD. *** *p* < 0.001.

As shown in Figure 7, the percentage of apoptotic cells increases with the length of the incubation time. The untreated cells which served as the control in the experiment exhibited the least percentage of necrotic cells, with a value of 0.08 ± 0.02%. During the first 24 h treatment, the percentage of necrotic cells rose to 2.31 ± 0.25%, whereas after 48 h, the percentage of necrotic cells climbed to 4.58 ± 0.14%. The treated cells incubated for 72 h, displayed a drop in the percentage of necrotic cells, with a value of 0.16 ± 0.01%. 

As expected, the untreated cells exhibited the highest value of viable cells, 99.6 ± 0.13%, indicating that the majority of the cells were still healthy and visible. Following treatment with raphasatin for 24, 48, and 72 h, the percentage of viable cells decreased significantly to a value of 54.26 ± 1.73%, 31.97 ± 1.03%, and 2.09 ± 0.24%, respectively. In the early apoptosis stage, the control incubations contained the lowest number of cells, 0.04 ± 0.02%, which was elevated to 7.68 ± 0.41%, following exposure to raphasatin for 24 h. Following the 48 h treatment, cell numbers declined to 5.12 ± 0.11%, and lastly, in contrast, the cell population increased to 22.90 ± 2.50%, at 72 h. Finally, in the late apoptosis stage, the fraction of apoptotic cells rose with the time of exposure to the isothiocyanate. The percentage of apoptotic cells was 35.45 ± 1.25%, 58.33 ± 0.87%, and 74.84 ± 2.37% at 24, 48, and 72 h, respectively; in the untreated MCF-7 cells, the number of apoptotic cells was 0.28 ± 0.09%.

### 2.4. Cell Cycle Analysis by Flow Cytometry

**Figure 8:**
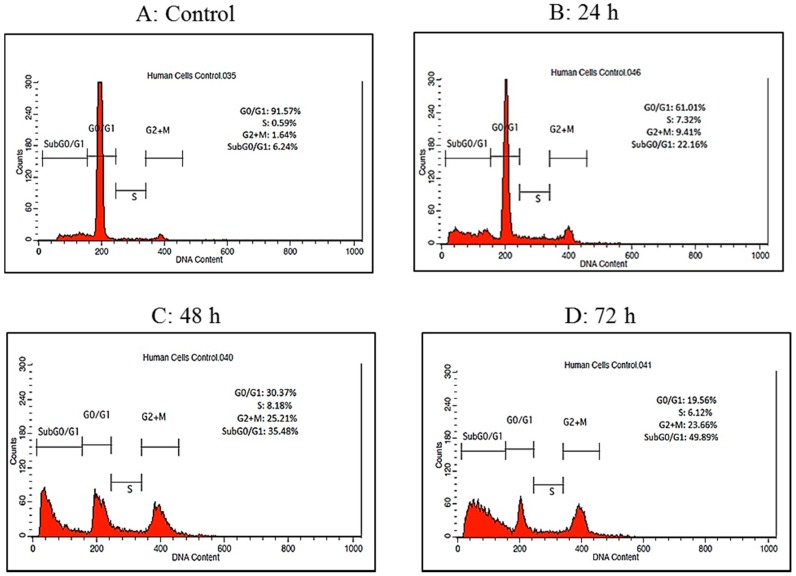
The time-dependent distribution of the MCF-7 cells exposed to raphasatin at the various stages of the cell cycle. MCF-7 cells were treated with IC_50_ concentration of raphasatin (9.84 µM) for 24 h (**B**), 48 h (**C**) and 72 h (**D**) compared with untreated cells (**A**). Raphasatin arrested the MCF-7 cells at the G_2_ + M phase as analyzed by flow cytometry. The data represent one of three independent experiments.

Cell cycle arrest analysis through flow cytometry was performed in order to evaluate whether the anti-proliferative effect of raphasatin in MCF-7 cells is related to changes in the cell cycle. The cellular distribution of the cells in different phases is illustrated in relation to the intracellular DNA content. The phases were divided into four, namely G_0_/G_1_, S, G_2_ + M, and Sub G_0_/G_1_ phase (Figure 8). 

**Figure 9:**
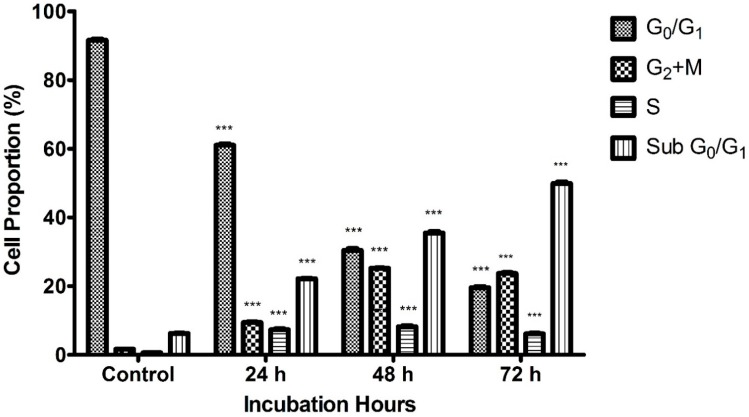
Time-dependent distribution of the raphasatin-treated MCF-7 cells for the different cell cycle stages. Cells were treated with 9.84 µM (IC_50_) concentration of raphasatin for 24, 48, and 72 h. The data comparing the proportion of cells between the untreated with the treated, in terms of cell cycle for 24, 48, and 72 h of incubation. The data expressed as mean ± SD. *** *p* < 0.001.

MCF-7 cells were treated with raphasatin (9.84 µM) for 24, 48 and 72 h. The percentage of cells in the Sub G_0_/G_1_ phase increased from 22.16 ± 0.16% to 35.48 ± 0.71% and 49.89 ± 0.71% at 24, 48 and 72 h respectively compared with the untreated cells (6.24 ± 0.18%) as shown in Figure 9. The percentage of cells in the G_0_/G_1_ phase showed a decreasing trend which was associated with an increase in percentage of cells at the G_2_ + M phase. The proportion of cells in the G_0_/G_1_ phase dropped to 61.01 ± 0.43%, 30.37 ± 0.58% and 19.56 ± 0.30% at 24, 48 and 72 h respectively, compared to the control (91.57 ± 0.40%). At the G_2_ + M phase, the percentage of cells recorded were 9.41 ± 0.34%, 25.21 ± 0.19% and 23.66 ± 0.54% at 24, 48 and 72 h respectively, compared to the control cells (1.64 ± 0.09%). Exposure to raphasatin led to the arrest of the cells at the G_2_ + M phase. Clearly, treatment of the MCF-7 cells with raphasatin inhibited cell cycle progression at the G_2_ + M phase so that it may be inferred that raphasatin impaired cell growth by arresting the cells at the G_2_ + M phase.

Finally, the lowest number of cells was observed at the end of the cell cycle. At the S phase, the proportion of cells recorded was 7.32 ± 0.51%, 8.18 ± 0.55% and 6.12 ± 0.39% at 24, 48 and 72 h respectively compared to the control cells (0.59 ± 0.08%).

## 3. Discussion

Extensive experimental studies have established that isothiocyanates, both aliphatic and aromatic, such as sulforaphane, erucin, and phenethyl isothiocyanate, owe their potent cancer chemopreventive activity to the fact that they act through a number of mechanisms, modulating favorably the initiation and promotion stages of carcinogenesis [18,22,23,24,25,26,27,28,29,30]. Raphasatin, present in substantial amounts in Daikon (Japanese white radish) as the glucosinolate glucoraphasatin, is consumed extensively in Japan [31]. Indeed, studies on rodents, conducted in Japan, demonstrated that raphasatin afforded protection against *N*-nitrosobenzylamine-induced esophageal cancer in rats [32] and *N*-nitrosobis(2-oxopropyl)amine-induced pancreatic cancer in hamsters [31], the effect being more pronounced when administered to the animals during the initiation stage, in comparison with the post-initiation stage. The reported antimutagenic in vitro activity of raphasatin [31,33], indicate that the isothiocyanate may protect DNA from the injury mediated by the reactive intermediates. This is generated following the bioactivation of chemical carcinogens and supports a beneficial role for isothiocyanate, at the initiation stage. This premise is reinforced by studies emanating from our laboratory where raphasatin, generated by the action of myrosinase on glucoraphasatin, was a potent inducer of enzyme systems involved in the deactivation of carcinogenic reactive intermediates, such as glutathione *S*-transferase, quinone reductase, and epoxide hydrolase, when incubated with precision-cut rat liver slices [20]. Similar observations have been made in vivo where rats maintained on a diet of a glucosinolate-rich Japanese Daikon extract had markedly higher levels of hepatic glutathione *S*-transferase activity, and to a lower extent of other detoxification enzymes [19]. As such, the antioxidant role of raphasatin is another key mechanism of its chemopreventive activity via antioxidant responsive elements (AREs) pathway, thus, the data on the antioxidant status would be beneficial in future. 

When administered to rats, raphasatin could also antagonize the chemically-induced carcinogenesis, when administered post-initiation, albeit less effectively, compared with when given during initiation, and the same workers also showed immunologically that the isothiocyanate could induce apoptosis, as well as impair cellular proliferation [32]. Moreover, hexane extracts of Daikon inhibited the proliferation in human cancer cells and stimulated apoptosis by influencing genes involved in apoptosis [14,15]. The commercial availability of raphasatin allowed us to extend these studies and evaluate, in detail, its potential to enhance apoptosis and arrest cell proliferation in human breast adenocarcinoma MCF-7 cells.

Apoptosis was monitored using the TUNEL assay, AO/PI, and DAPI staining, and by Annexin V-FITC/PI double-staining, followed by flow cytometry. In all assays raphasatin clearly elevated apoptosis, as was exemplified by the increased membrane blebbing, chromatin condensation, nuclear fragmentation, and the formation of micronuclei; furthermore, the effect was related to the time of exposure. These findings demonstrate that increased apoptosis is a viable mechanism contributing to the cancer chemopreventive effect of raphasatin.

The potential of raphasatin to perturb the cell cycle was assessed in the present study, using flow cytometry, in order to establish whether the anti-proliferative effect of this isothiocyanate is related to changes in the cell cycle. In a previous study by Papi et al. [15], conducted in CaCo-2 cells, an increase in the G_0_/G_1_ population was observed in the presence of raphasatin. In the present study, flow cytometry analysis revealed that raphasatin significantly arrested the cell cycle in the MCF-7 cells, at the G_2_ + M phase. The percentage of cells in the G_2_ + M phase increased, significantly, probably reflecting apoptosis induced by raphasatin, resulting in DNA degradation. Anticancer agents may modify the regulation of the cell-cycle machinery, resulting in an arrest of cells in various phases of the cell cycle, thus, decreasing the cancerous cells growth and proliferation [34].

It is pertinent to point out that raphasatin has caused a marked increase in apoptosis and arrest in cell cycle, at a concentration lower than 10 µM. Although to our knowledge the pharmacokinetic behavior of raphasatin has not been investigated, studies performed with other isothiocyanates, such as sulforaphane and phenethyl isothiocyanate, in rodents, noted that such concentrations in the plasma may be achieved following exposure to doses simulating the human dietary intake [35,36,37,38,39,40,41,42]. However, the distribution of isothiocyanates to tissues, following exposure to dietary doses, has not been evaluated, so that the concentration of the parent compound in the breast cell has not been defined. It is pertinent to point out that intracellular concentrations of isothiocyanates, including their dithiocarbamate metabolites, may be much higher than those encountered in the plasma [43,44]. Moreover, cruciferous vegetables strains with high glucosinolate content may be developed to increase the level of exposure to isothiocyanates [45].

## 4. Materials and Methods

### 4.1. Isolation of Glucoraphasatin (GRH)

GRH was isolated from the sprouts of *Raphanus sativus* L. (Daikon), as previously described [15]. Briefly, GRH was isolated from the seven-day-old *Raphanus sativus* L. sprouts which were freeze-dried through two sequential steps, including ion exchange and size exclusion chromatography. GRH (10 mg/mL) was dissolved in water and kept in a freezer, at −20 °C. Raphasatin was produced through myrosinase-catalyzed hydrolysis [46]. In each experiment, 5 µL of myrosinase (0.3 units/mL), isolated from the seeds of *Sinapsis alba* L., was added into the cell culture medium to generate raphasatin. One myrosinase unit is defined as the amount of enzyme capable of hydrolyzing 1 µmol sinigrin, per min, at pH 6.5 and 37 °C.

### 4.2. Cell Viability

MCF-7 (Human breast adenocarcinoma) cell line was obtained from the American Type Culture Collection (ATCC, Manassasa, VA, USA). The cells were cultured in a flask containing RPMI 1640 (Sigma-Aldrich, Munich, Germany) supplemented with 10% FBS (fetal bovine serum, Sigma-Aldrich, Munich, Germany), 1% of antibiotics (Streptomycin & Penicillin) (Sigma-Aldrich, Munich, Germany), and incubated in a humidified incubator supplemented with 5% CO_2_, at a temperature of 37 °C. Cell viability was determined using the MTT assay. The MCF-7 cells were seeded in 96-well flat-bottomed tissue culture plates, at a concentration of 1 × 10^5^ cells/well, and allowed to grow for 24 h. Upon 50–70% of confluency, the cells were treated with GRH at concentrations ranging from 0–100 µM. Five µL of myrosinase (0.3 units/mL) (Sigma Aldrich, St. Louis, MO, USA) was added into each well to hydrolyze the GRH into the raphasatin, before the cell treatment. The cells were then incubated for 24, 48, and 72 h. At the end of the incubation, 20 µL (5 mg/mL) of the MTT reagent (Sigma-Aldrich, Munich, Germany) was added into each well, and incubated for 3 h, at 37 °C. The medium was removed and 100 µL of dimethyl sulfoxide (Sigma-Aldrich, St. Louis, MO, USA) and was added into each well, resulting in the solubilization of the formazan salt. The absorbance was measured at 570 nm, using an ELISA reader (TECAN, Männedorf, Zürich, Switzerland) and the IC_50_ (50% inhibitory concentration) was calculated [47]. 

### 4.3. Acridine Orange/Propidium Iodide Staining

The MCF-7 cells were seeded in 25 cm^2^ culture flasks, at a concentration of 1 × 10^6^ cells/mL, and incubated for 24 h. Following incubation, the cells were treated with 9.84 µM (IC_50_) concentration of raphasatin. After the treatment, the supernatant was collected and transferred into a falcon tube, whereas, the remaining cells in the flask were trypsinized and collected, using a scraper. The cells were mixed with 2 mL PBS, resuspended, and mixed with the supernatant in the falcon tube. The cells were centrifuged at 300× *g* for 5 min. The supernatant was discarded and cells were washed again, using 5 mL PBS. The centrifugation step was repeated and the falcon tube was put on ice. One ml Acridine Orange/Propidium Iodide (AO/PI) solution (Sigma-Aldrich, St. Louis, MO, USA) was diluted with PBS (1 mL), the process being carried out under dark conditions, due to the sensitivity of both solutions to light. AO and PI solutions (10 mg/mL of each) were transferred into an Eppendorf tube and the mixture was resuspended, repeatedly, until a homogeneous solution was obtained. An aliquot of the cell preparation (10 µL) was mixed with the prepared AO/PI solution. The mixture (10 µL) was transferred on to a slide and viewed under a UV-fluorescent microscope, using 20× objective lens (Axio Scope A1, Zeiss, Oberkochen, Germany).

### 4.4. DAPI Staining

Cells were grown on coverslips. After a 24 h treatment with raphasatin, the cells were fixed with 4% paraformaldehyde and permeabilized with Triton X-100 (0.1% in PBS). Cells were stained with 2.5 mg/mL DNA dye, 4′,6-diamidino-2-phenylindole (DAPI) (Sigma-Aldrich, St. Louis, MO, USA) in PBS, for 30 min, at 20 °C, and examined by fluorescence microscopy. Cells with condensed or fragmented nuclei were counted on the adjacent four fields, of each coverslip. The percentage of cells with chromatin condensation and nuclear fragmentation was determined in triplicates, for each treatment, and compared with the controls [47]. Morphological changes were viewed using a UV-fluorescent microscope (Axio Scope A1, Zeiss, Oberkochen, Germany) employing a 20× objective lens.

### 4.5. DeadEnd Colorimetric TUNEL Assay

The MCF-7 cells were seeded at a concentration of 1 × 10^6^ cells/mL, in 25 cm^2^ culture flasks, and incubated for 24 h. The terminal deoxynucleotidyl transferase dUTP nick end labelling (TUNEL) assay was carried out using the DeadEnd^TM^ colorimetric apoptosis detection system (Promega, Madison, WI, USA), according to the manufacturer’s protocol. After the treatment with raphasatin, the cells were harvested and grown onto poly-l-lysine slides, for 6 h. Slides were immersed in 4% paraformaldehyde and then twice in PBS. Subsequently, slides were immersed in 0.2% Triton^®^ X-100 in PBS for 5 min and washed twice in PBS. Equilibration Buffer (100 µL) was added on to the slides, followed by 100 μL of terminal deoxynucleotidyl transferase (TdT) reaction mix. Slides were covered with plastic coverslips and incubated for 60 min, at 37 °C, in a humidified chamber. After incubation, slides were immersed in 2× Saline Sodium Citrate (SSC), for 15 min, followed by 0.3% hydrogen peroxide for 3–5 min. Thereafter, 100 μL Streptavidin Peroxidase (diluted 1:500 in PBS) was added, and slides were incubated for a further 30 min, at room temperature. 3,3′-Diaminobenzidine (DAB) (100 µL) was added until a light brown background appeared; slides were then immersed several times in deionized water. Lastly, slides were mounted in permanent mounting medium and staining was observed, under a light microscope (Microscope Compound Olympus) (Olympus, Tokyo, Japan) using a 20× objective lens [47].

### 4.6. Annexin V-FITC/PI by Flow Cytometry

Annexin V-FITC assay was carried out using the Annexin V-FITC assay kit (Sigma-Aldrich, St. Louis, MO, USA), according to the protocols provided. The MCF-7 cells at a concentration of 1 × 10^6^ cells/mL were seeded in 25 cm^2^ culture flasks, and incubated for 24 h. At the end of the incubation, the cells were treated with the IC_50_ concentrations of raphasatin and incubated for 24, 48, and 72 h. After the treatment, the supernatant was collected and transferred into a falcon tube. The remaining cells in the flask were trypsinized, using 1 mL trypsin, incubated for 5 min, and the cells were collected and transferred into a falcon tube. The cells were mixed with 2 mL PBS, resuspended, and mixed with the supernatant in the falcon tube. The cells were centrifuged at 300× *g*, for 5 min. The supernatant was discarded and the precipitate was washed again, using 5 mL PBS. The centrifugation step was repeated and the falcon tube was put into an ice box. Flow cytometric analysis was carried out using a FACS Canto II Becton–Dickinson (Franklin Lakes, NJ, USA) flow cytometer, by analyzing at least 10,000 cells per sample. The binding buffer supplied by the manufacturer was used to bring the reaction volume to at least 500 μL, for the flow cytometry analysis. Unstained cells were classified as viable cells; cells stained for Annexin-V were early apoptotic; cells stained with both Annexin-V and PI were late apoptotic; cells stained with PI were indicated as necrotic cells [47]. Results are presented as percentage (%) of the total number of cells. 

### 4.7. Cell Cycle Arrest Analysis by Flow Cytometry

Cell cycle arrest evaluation was carried out using a Cell Cycle Arrest Detection Kit (Sigma-Aldrich, Becton Dickinson, Franklin Lakes, NJ, USA), according to the protocol provided. MCF-7 cells at a concentration of 1 × 10^6^ cells/mL were harvested and transferred into 17 × 100-mm tubes. The cells were centrifuged for 5 min at 300× *g*, at room temperature, and the supernatant was aspirated. Buffer solution (1 mL) was added and the cells were resuspended by gentle vortexing, at low speed. The concentration was adjusted to 1.0 × 10^6^ cells/mL, with buffer solution. The cell suspensions were centrifuged at 300× *g* for 5 min at room temperature, and the supernatant was decanted carefully. Solution A (containing trypsin buffer), at a volume of 250 µL, was added to each tube and gently mixed by hand tapping the tube. Solution A was allowed to react for 10 min at room temperature. Two hundred µl of Solution B (containing trypsin inhibitor and RNase buffer) was added into each tube and gently mixed by hand tapping, and a further 10 min incubation was carried out, at room temperature. Cold Solution C (containing propidium iodide stain solution, 200 µL) was added into each tube. The solution was mixed, as above, and incubated once again for 10 min, on ice, in the dark. The tubes were finally capped and stored at 2–8 °C, in the dark, until flow cytometric analysis was performed. The samples were tested within 3 h, following the addition of Solution C. After storage, the samples in the tubes were mixed by hand tapping, to resuspend the cells. The cells were then analyzed using the FACS Canto II Becton-Dickinson (Franklin Lakes, NJ, USA) flow cytometer, by analyzing at least 10,000 cells per sample. The percentage of cells in G1, S, and G2 phases were analyzed by the ModFit LT software (Verity Software House, Topsham, ME, USA).

### 4.8. Statistical Analysis

Data are expressed as mean ± standard deviation (SD). Statistical evaluation was performed by two-way ANOVA, using Dunnett’s test. Differences were considered significant at *p* < 0.05. All the samples were measured in triplicates.

## 5. Conclusions

In conclusion, the present studies performed in the MCF-7 cells have established that raphasatin, at concentrations potentially achieved in the plasma, following dietary intake, stimulates apoptosis and impairs cell proliferation, and that these mechanisms may be integral to its cancer chemopreventive activity.

## Figures and Tables

**Table 1:** Cytotoxicity of GRH, raphasatin, and paclitaxel in human breast adenocarcinoma MCF-7 cells, following incubation for 24, 48, and 72 h, using 3-(4,5-dimethylthiazol-2-yl)-2,5-diphenyltetrazolium bromide (MTT) assay. Data are expressed as mean ± standard deviation (SD) of three independent experiments.

Compound	IC_50_		
	24 h	48 h	72 h
GRH	>100 µM	>100 µM	>100 µM
Raphasatin	9.84 ± 0.55 µM	9.64 ± 0.24 µM	5.61 ± 0.23 µM
Paclitaxel	7.53 ± 0.68 nM	5.21 ± 0.28 nM	5.09 ± 0.07 nM

## References

[1] Gerl R., Vaux D.L. (2005). Apoptosis in the development and treatment of cancer. Carcinogenesis.

[2] Dyer N. (1999). Venom: Miracle Medicine. Sci. World.

[3] Benjamin C.W., Hiebsch R.R., Jones D.A. (1998). Caspase activation in MCF-7 cancer cells responding to etoposide treatment. Mol. Pharm..

[4] Hisham A.N., Yip C.H. (2003). Spectrum of breast cancer in Malaysian women: Overview. World J. Surg..

[5] Yip C.H., Taib N.A., Mohamed I. (2006). Epidemiology of breast cancer in Malaysia. Asian Pac. J. Cancer Prev..

[6] Omar Z.A., Ali Z.M., Tamin N.S.I. (2006). Malaysian Cancer Statistics-Data and Figure Peninsular Malaysia 2006.

[7] Podsedek A. (2007). Natural antioxidants and antioxidant capacity of Brassica vegetables: A review. Swiss Soc. Food Sci. Technol..

[8] Zhang N.Q., Ho S.C., Mo X.F., Lin F.Y., Huang W.Q., Luo H., Zhang C.X. (2018). Glucosinolate and isothiocyanate intakes are inversely associated with breast cancer risk: A case–control study in China. Br. J. Nutr..

[9] Lin T., Zirpoli G.R., McCann S.E., Moysich K.B., Ambrosone C.B., Tang L. (2017). Trends in cruciferous vegetable consumption and associations with breast cancer risk: A case-control study. Curr. Dev. Nutr..

[10] Hayes J.D., Kelleher M.O., Eggleston I.M. (2008). The cancer chemopreventive actions of phytochemicals derived from GLs. Eur. J. Nutr..

[11] Steinbrecher A., Linseisen L. (2009). Dietary intake of individual GLs in participants of the EIPC-Heidelberg cohort study. Ann. Nutr. Metab..

[12] Fausta N., Mariateresa M., Guido L., Cristina S. (2014). Glucosinolates redox activities: Can they act as antioxidants?. Food Chem..

[13] Talalay P., Fahey J.W. (2001). Phytochemicals from cruciferous plant protect against cancer by modulating carcinogen metabolism. J. Nutr..

[14] Beevi S.S., Mangamoori L.N., Subathra M., Edula J.R. (2010). Hexane extract of *Raphanus sativus* L. roots inhibits cell proliferation and induces apoptosis in human cancer cells by modulating genes related to apoptotic pathway. Plant Foods Hum. Nutr..

[15] Papi A., Orlandi M., Bartolini G., Barillari J., Iori R., Paolini M., Ferroni F., Grazia Fumo M., Pedulli G.F., Valgimigli L. (2008). Cytotoxic and antioxidant activity of 4-methylthio-3-butenyl isothiocyanate from *Raphanus sativus* L. (Kaiware Daikon) sprouts. J. Agric. Food Chem..

[16] Fimognari C., Lenzi M., Hrelia P. (2008). Chemoprevention of cancer by isothiocyanates and anthocyanins: Mechanisms of action and structure-activity relationship. Curr. Med. Chem..

[17] Taatjes D.J., Sobel B.E., Budd R.C. (2008). Morphological and cytochemical determination of cell death by apoptosis. Histochem. Cell Biol..

[18] Abdull Razis A.F., Konsue N., Ioannides C. (2018). Isothiocyanates and xenobiotic detoxification. Mol. Nutr. Food Res..

[19] Abdull Razis A.F., De Nicola G.R., Pagnotta E., Iori R., Ioannides C. (2013). A glucosinolate-rich extract of Japanese Daikon perturbs carcinogen-metabolizing enzyme systems in rat, being a potent inducer of hepatic glutathione *S*-transferase. Eur. J. Nutr..

[20] Razis A.F.A., De Nicola G.R., Pagnotta E., Iori R., Ioannides C. (2012). 4-Methylsulfanyl-3-butenyl isothiocyanate derived from glucoraphasatin is a potent inducer of rat hepatic phase II enzymes and a potential chemopreventive agent. Arch. Toxicol..

[21] Kntayya S.B., Ibrahim M.D., Mohd Ain N., Iori R., Ioannides C., Abdull Razis A.F. (2018). Induction of apoptosis and cytotoxicity by isothiocyanate sulforaphene in human hepatocarcinoma HepG2 cells. Nutrients.

[22] Arumugam A., Razis A.F.A. (2018). Apoptosis as a mechanism of the cancer chemopreventive activity of glucosinolates: A review. Asian Pac. J. Cancer Prev..

[23] Razis A.F.A., Konsue N., Ioannides C. (2015). Inhibitory effect of phenethyl isothiocyanate against benzo[a]pyrene-induced rise in CYP1A1 mRNA and apoprotein levels as its chemopreventive properties. Asian Pac. J. Cancer Prev..

[24] Razis A.F.A., Mohd Noor N., Konsue N. (2014). Induction of epoxide hydrolase, glucuronosyl transferase, and sulfotransferase by phenethyl isothiocyanate in male Wistar albino rats. Biomed. Res. Int..

[25] Razis A.F.A., Noor N.M. (2013). Sulforaphane is superior to glucoraphanin in modulating carcinogen-metabolising enzymes in Hep G2 cells. Asian Pac. J. Cancer Prev..

[26] Razis A.F.A., Hanlon N., Soltys E., Krizova V., Iori R., Plant K.E., Ioannides C. (2012). The naturally occurring aliphatic isothiocyanates sulforaphane and erucin are weak agonists but potent non-competitive antagonists of the aryl hydrocarbon receptor. Arch. Toxicol..

[27] Abdull Razis A.F., Konsue N., Dervetzoglou M., Plant K.E., Plant N., Ioannides C. (2012). Phenethyl isothiocyanate, a naturally occurring phytochemical, is an antagonist of the aryl hydrocarbon receptor. Mol. Nutr. Food Res..

[28] Abdull Razis A.F., Iori R., Ioannides C. (2011). The natural chemopreventive phytochemical R-sulforaphane is a far more potent inducer of the carcinogen-detoxifying enzyme systems in rat liver and lung than the S-isomer. Int. J. Cancer.

[29] Razis A.F.A., Bagatta M., De Nicola G.R., Iori R., Ioannides C. (2011). Induction of epoxide hydrolase and glucuronosyl transferase by isothiocyanates and intact glucosinolates in precision-cut rat liver slices: Importance of side-chain substituent and chirality. Arch. Toxicol..

[30] Okamura T., Umemura T., Inoue T., Tasaki M., Ishii Y., Nakamura Y., Park E.Y., Sato K., Matsuo T., Okamoto S. (2013). Chemopreventive effects of 4-methylthio-3-butenyl isothiocyanate (*Raphasatin*) but not curcumin against pancreatic carcinogenesis in hamsters. J. Agric. Food Chem..

[31] Nakamura Y., Iwahashi T., Tanaka A., Koutani J., Matsuo T., Okamoto S., Sato K., Ohtsuki K. (2001). 4-(Methylthio)-3-butenyl isothiocyanate, a principal antimutagen in daikon (*Raphanus sativus*; Japanese white radish). J. Agric. Food Chem..

[32] Suzuki I., Cho Y.M., Tadashi H., Takeshi T., Jun-ichi A., Yasushi N., Eun Y.P., Sasaki A., Nakamura T., Okamoto S. (2016). 4-Methylthio-3-butenyl isothiocyanate (*Raphasatin*) exerts chemopreventive effects against esophageal carcinogenesis in rats. J. Toxicol. Pathol..

[33] Ben Salah-Abbès J., Abbès S., Ouanes Z., Abdel-Wahhab M.A., Bacha H., Oueslati R. (2009). Isothiocyanate from the Tunisian radish (*Raphanus sativus*) prevents genotoxicity of Zearalenone in vivo and in vitro. Mutat. Res..

[34] Shah M.A., Schwartz G.K. (2005). Cyclin-dependent kinases as targets for cancer therapy. Cancer Chem. Biol. Response Mod..

[35] Hanlon N., Okpara M., Coldham N., Sauer M.J., Ioannides C. (2008). Modulation of rat hepatic and pulmonary cytochromes P450 and Phase II enzyme systems by erucin, an isothiocyanate structurally related to sulforaphane. J. Agric. Food Chem..

[36] Hanlon N., Coldham N., Sauer M.J., Ioannides C. (2008). Up-regulation of the CYP1 family in rat and human liver by the aliphatic isothiocyanates erucin and sulforaphane. Toxicology.

[37] Hanlon N., Coldham N., Gielbert A., Kuhnert N., Sauer M.J., King L.J., Ioannides C. (2008). Absolute bioavailability and dose-dependent pharmacokinetic behaviour of dietary doses of the chemopreventive isothiocyanate sulforaphane in the rat. Br. J. Nutr..

[38] Hanlon N., Coldham N., Sauer M.J., Ioannides C. (2009). Modulation of rat pulmonary carcinogen-metabolising enzyme systems by the isothiocyanates erucin and sulforaphane. Chem. Biol. Interact..

[39] Konsue N., Ioannides C. (2008). Tissue differences in the modulation of rat cytochromes P450 and phase II conjugation systems by dietary doses of phenethyl isothiocyanate. Food Chem. Toxicol..

[40] Konsue N., Ioannides C. (2010). Differential response of four human livers to modulation of phase II enzyme systems by the chemopreventive phytochemical phenethyl isothiocyanate. Mol. Nutr. Food Res..

[41] Konsue N., Ioannides C. (2010). Modulation of carcinogen-metabolising cytochromes P450 in human liver by the chemopreventive phytochemical phenethyl isothiocyanate, a constituent of cruciferous vegetables. Toxicology.

[42] Konsue N., Kirkpatrick J., Kuhnert N., King L.J., Ioannides C. (2010). Repeated oral administration modulates the pharmacokinetic behaviour of the chemopreventive agent phenethyl isothiocyanate in rats. Mol. Nutr. Food Res..

[43] Zhang Y. (2000). Role of glutathione in the accumulation of anticarcinogenic isothiocyanates and their glutathione conjugates by murine hepatoma cells. Carcinogenesis.

[44] Zhang Y. (2001). Molecular mechanism of rapid cellular accumulation of anticarcinogenic isothiocyanates. Carcinogenesis.

[45] Traka M.H., Saha S., Huseby S., Kopriva S., Walley P.G., Barker G.C., Moore J., Mero G., van den Bosch F., Constant H. (2013). Genetic regulation of glucoraphanin accumulation in Beneforté broccoli. New Phytol..

[46] Barillari J., Cervellati R., Paolini M., Tatibouet A., Rollin P., Iori R. (2005). Isolation of 4 methylthio-3-butenyl GLs from *Raphanus sativus* L. sprouts (Kaiware-Daikon) and its redox properties. J. Agric. Food Chem..

[47] Ismail A.A., Ahmad B.A., Mohd A.S., Rasedee A., Suvitha S., Behnam K., Mohamed Y.I., Manal M.E., Taha S.I.A., Hapipah M.A. (2013). Dentatin isolated from *Clausena excavata* induces apoptosis in MCF-7 cell line through the intrinsic pathway with involvement of NF-*κ*B signalling and G_0_/G_1_ cell cycle arrest: A bioassay guided approach. J. Ethnopharmacol..

